# A comparison of glycemic parameters and their relationship with C-peptide and Proinsulin levels during partial remission and non-remission periods in children with type 1 diabetes mellitus - a cross-sectional study

**DOI:** 10.1186/s12902-021-00681-1

**Published:** 2021-01-23

**Authors:** Gül Yeşiltepe-Mutlu, Merve Çapacı, Ecem Can, Tuğba Gökçe, Gizem Bayrakçı, Serra Muradoğlu, Said İncir, Esra Papatya Çakır, Şükrü Hatun

**Affiliations:** 1 Department of Pediatric Endocrinology and Diabetes, Koç University Hospital, Topkapı, Zeytinburnu, 34010 Istanbul, Turkey; 2 Koç University School of Medicine, Istanbul, Turkey; 3 Department of Biochemistry, Koç University Hospital, Istanbul, Turkey; 4 Department of Pediatric Endocrinology, Bakirköy Dr Sadi Konuk Training and Research Hospital, Istanbul, Turkey

**Keywords:** Type 1 diabetes, Partial remission, C-peptide, Proinsulin

## Abstract

**Background:**

Currently, there is a lack of data relating to glycemic parameters and their relationship with C-peptide (CP) and proinsulin (PI) during the partial remission period (PRP) in type 1 diabetes mellitus (T1D). The aim of this study was to evaluate glycemic parameters in children with T1D who are in the PRP using intermittently scanned continuous glucose monitoring systems (isCGMS) and to investigate any relationships between CP and PI levels.

**Methods:**

The study included 21 children who were in the PRP and 31 children who were not. A cross-sectional, non-randomized study was performed. Demographic, clinical data were collected and 2 week- isCGMS data were retrieved.

**Results:**

The Serum CP showed a positive correlation with time-in-range in the PRP (p:0.03), however PI showed no correlations with glycemic parameters in both periods. The Serum CP and PI levels and the PI:CP ratio were significantly higher in the PRP group than in the non-PRP group. In the non-PRP group, the PI level was below 0.1 pmol/L (which is the detectable limit) in only 2 of the 17 cases as compared with none in the PRP group. Similarly, only 2 of the 17 children in the non-PRP group had CP levels of less than 0.2 nmol / L, although both had detectable PI levels. Overall time-in-range (3. 9-1.0 mmol/L) was significantly high in the PRP group. In contrast, the mean sensor glucose levels, time spent in hyperglycemia, and coefficient of variation levels (32.2vs 40.5%) were significantly lower in the PRP group.

**Conclusions:**

Although the mean glucose and time in range during the PRP was better than that in the non-PRP group, the glycemic variability during this period was not as low as expected. While the CP levels showed an association with TIR during the PRP, there was no correlation between PI levels and glycemic parameters. Further studies are needed to determine if PI might prove to be a useful parameter in clinical follow-up.

## Background

The transient recovery period of beta-cell function, after initiation of insulin therapy in patients with newly diagnosed type 1 diabetes mellitus, is referred to as the ‘honeymoon’ or partial remission phase (PRP). The clinical significance of this period lies in the maintenance of glycemic control characterized by a reduction in insulin requirements. Previous controlled trials on the natural course of beta cell function recovery reported the prevalence of remission as 60%, [1] with a duration ranging from 1 month to as long as 13 years [2]. To achieve better glycemic control throughout the remission phase, several studies focused on the factors influencing the natural course of remission, such as severity of presentation, age at diagnosis, gender, and effects of autoantibodies. Nevertheless, the glycemic parameters and variability in these parameters during the remission period remain unknown.

PRP is defined as an insulin dose-adjusted hemoglobin A1c (HbA1c) (IDAA1c) of equal to or less than 9, where IDAA1c is equal to the sum of HbA1c (% and mmol/mol) and 4 times the insulin dose (units/kg/day) [3]. However, this definition based on insulin doses and HbA1c is not sufficient in terms of glycemic variability, insulin sensitivity, and episodes of hypoglycemia and hyperglycemia. Furthermore, there are no definite cut-off values for C-peptide (CP) and proinsulin (PI) levels for the definition of the PRP in type 1 diabetes (T1D). However, the measurement of the CP levels for the definition of the remission period has been established and is a laborious and expensive process [3]. Additionally, an IDAA1C level of 9 has been shown to correspond to a substantially increased predicted stimulated C-peptide (CP) level of 300 pmol / l [3].

Normal insulin biosynthesis is a multi-step process, beginning with a pre-prohormone, pre-pro-insulin, which is then converted to proinsulin (PI). PI becomes incorporated into a new “immature” beta-granule, where it is subsequently cleaved into insulin and CP via prohormone convertases (PCSK1, PCSK2, and carboxypeptidase E) [4]. It has been shown that the PI to CP ratio (PI: CP) increases in individuals at risk of diabetes and at the time of diagnosis of diabetes. On the other hand, Watkins et al. examined PI levels at the time of T1D diagnosis, shortly after diagnosis, and during the PRP and found that the PI level was higher during the PRP than that at the time of diagnosis [5]. However, the importance of neither CP nor PI levels in clinical practice and their effects on glycemic parameters have not been investigated in detail.

In the past, only the HbA1C level was taken into account for defining glycemic control in diabetes. However, new glycemic parameters have emerged in the light of recent advances in diabetes technology. Currently, the definition of RP is still based only on HbA1C levels and insulin doses and there are few studies evaluating other glycemic parameters in this period. In the light of recent technological developments, this study aimed to evaluate the glycemic parameters in children with T1D who are in PRP and compare them with those who are not in this phase using intermittently scanned continuous glucose monitoring systems (isCGMS). The study also aimed to investigate the relationships between PI and CP levels and glycemic parameters in order to draw comparisons between the PRP and non-PRP periods.

## Methods

### Subjects

**Fig. 1:**
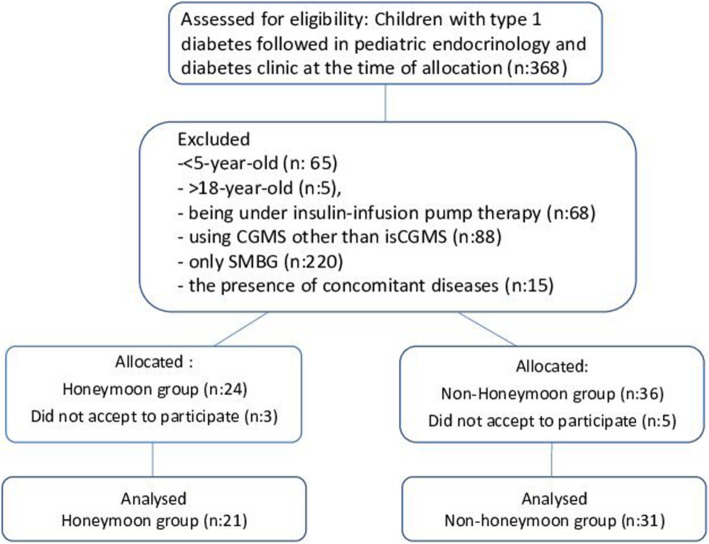
Study flow diagram

The study was conducted between March 2018 and 2019 and included 21 children with T1D who were in PRP (the PRP group) and 31 children with T1D who had been diagnosed at least 2 years before the enrollment and who were not in RP (the non-PRP group). The inclusion criteria required an age of 5 to 18 years and being under multiple-dose insulin injection therapy and isCGMS. The exclusion criteria were the presence of concomitant diseases that could influence metabolic control, the use of an insulin infusion pump and having HbA1C level above 9% (75 mmol/mol) (A consort flow diagram is showed in Fig. 1).

### Study design

A cross-sectional, non-randomized study was performed. The study protocol was approved by the Koç University Committee on Human Research (reference number:2018.022.IRB1.004). Written consent was obtained from the parents along with assent from the adolescents as required by the local institutional review board regulations.

A partial remission phase was defined as an insulin dose adjusted HbA1c (IDAA1c) of equal to or less than 9 [1]. Demographic, socioeconomic, and clinical data were collected from medical records and from interviews with the participants and parents. Also, 2 weeks’ isCGMS data were retrieved using the Free-Style Libre software in the outpatient pediatric endocrinology clinic. In addition to the software report, the raw data were used for statistical analysis and assessed according to continuous glucose monitoring systems (CGMS) consensus report [6]. Random (mostly non-fasting) serum samples were collected for measurement of CP and PI during the routine follow-up visits.

### Laboratory analyses

For the measurement of CP and PI, samples were collected into dry tubes and centrifuged promptly (3500 g) for 10 min at + 4 °C. The sera were separated in aliquots and frozen immediately at − 80 °C. Serum CP and PI levels were determined by competitive ELISA using commercial kits (DRG Instruments GmBH, Germany). Intra- and inter-CVs were 6.54 and 9.33% for CP, and 4.3 and 6.8% for PI, respectively. The detection limit was 0.1 pmol/L for PI [7]. HbA1c levels were analyzed immediately after collecting blood samples into K_2_-EDTA-added tubes on an ADAMS A1c Lite HA-8380 V analyzer (Arkray) using the HPLC (Reversed-phase cation-exchange liquid chromatography) method. Sustained levels of CP were considered to be consistent readings greater than 0.2 nmol/l [8].

### Statistical analyses

The sample size calculation was made using OpenEpi Statistical Software version 3.01 [9]. Type 1 error of the study is α = 0,05, the power of the study is 1-β = 0,80, and the number of samples was calculated as 18 per group. A total of at least 40 people had to be included in the study, 20 per group, with an appendix of approximately 10 % loss. The other statistical analyses were conducted using SPSS statistical software version 22 (USA). Descriptive statistics included means, standard deviation (SD), median, interquartile range, and proportions when appropriate. Correlations between CP, PI and glycemic parameters were computed using Spearman’s correlation coefficient. For continuous variables, mean ± standard deviation and median values were used for variables with and without normal distribution, respectively. For continuous variables, the Student’s t-test was used for normally distributed data and the Mann-Whitney U-test was used for data without normal distribution.

## Results

The two groups were similar in terms of age and gender. As expected, the mean diabetes duration, daily insulin dose, HbA1c, IDDA1c, estimated HbA1c levels were significantly lower in the PRP group compared with the non-PRP group. However, the serum CP and PI levels and the PI:CP ratio were significantly higher in the PRP group than those in the non-PRP group. The demographic characteristics and laboratory findings of the two groups are given in Table 1. A total of 17 children in each group agreed to give serum samples for the measurement of PI and CP. In the non-PRP group, PI level was below 0.1 pmol/L (which is the detectable limit) in only 2 of the 17 cases as compared with none in the PRP group. Similarly, only 2 of the 17 children in the non-PRP group had CP levels of less than 0.2 nmol / L, although both had detectable PI levels.

**Table 1.** The demographic characteristics and laboratory findings of the participants

	PRP group (n:21)	Non-PRP group (n:31)	*P* value
Age (year)	9.3±3(5-13.7)	8.9±2.6(5-13.8)	0.67
Gender (female/male)	9/12	16/15	0.259
Diabetes duration (years)	0.57±0.5(0.04-1.7)	4.3±1.5(2.2-8.7)	**<0.001**
Daily insulin dose (u/kg)	0.35±0.11(0.13-0.5)	0.81±0.16(0.55-1.25)	**<0.001**
HbA1c (%) (mmol/mol)	6.4(46)±0.6(-17)((5.2(33)-7.6(60))	7.2(55)±0.6(-17)((6.2(44)-8.3(67))	**<0.001**
IDDAA1C (%)(mmol/mol)	7.8(62)±0.7(0.16)((6.8(51)-8.9(74))	10.3(89)±0.8(-0.15)((8.6(70)-11.9(107))	**<0.001**
Estimated HbA1c (%)	6.5±0.8(4.6-8.1)	7.6 ±0.9(6.1-9.7)	**0.006**
CP level (pmol/L) (n:17)	739.8±537.8(288-2582)	465.6±478.9(102.6-1854)	**0.002**
PI level (pmol/L)(n:17)	3.9±3.7(0.64-12.1)	0.48±0.33(0.04-1.3)	**<0.001**
PI:CP ratio	0.55 0.0055 ± 0.004(0.00085-0.15)	0.13 0.0013 ± 0.001(0.00016-0.003)	**<0.001**

*IDDAA1C: Insulin Dose Adjusted HbA1C, CP* C-peptide*, PI* Proinsulin*, PI:CP Proinsulin to C-peptide ratio*

When the glycemic parameters were compared in the two groups (Table 2), overall time-in-range, day-time and night-time levels were significantly higher in the PRP group. In contrast, the mean sensor glucose levels (overall, day-time and night-time), the mean time spent in level 1 and level 2 hyperglycemia overall, day-time and night-time, the median time spent in level 1 hypoglycemia (< 3.9 mmol/L) day-time, and the mean coefficient of variation (CV) and SD levels were all significantly lower in the PRP group.

**Table 2:** Glycemic parameters of the participants

	PRP group (n:21)	Non-PRP group (n:31)	*P* value
Sensor glucose overall (mean) (mmol/L)	7.6 ± 1.2(4.3-10.2)	9.4 ± 1.3(7.2-12.8)	**<0.001**
Sensor glucose (mean) Day-time (06-00) (mmol/L)	7.6 ± 1.2 (1.2-10)	9.4 ± 1.3(7.3-12.8)	**0.001**
Sensor glucose (mean) Night-time(00-06)(mmol/L)	7.5 ± 1.6(4.2-11)	9.4 ± 1.9(6.2-13.7)	**0.002**
CV overall (mean) %	32.3±5 (21-44)	40.5±6 (32-56)	**<0.001**
CV day-time (mean) %	32.1±4.7(21-42)	40.6±6(33-54)	**<0.001**
CV night-time(mean) %	29±5 (19-45)	38.2 ±1(24-64)	**<0.001**
SD overall (mean)(mmol/L)	2.5 ± 0.6(1-3.7)	3.8 ± 0.7(2.7-5.2)	**<0.001**
SD day-time (mean)(mmol/L)	2.5 ± 0.7 (1-3.6)	3.8 ± 0.6(2.6-5.1)	**<0.001**
SD night-time (mean) (mmol/L)	2.2 ± 0.7 (0.9-4.1)	3.5 ± 1(2.2-6)	**<0.001**
Overall time-in-range (3.9-10 mmol/L (%)	76.2±11.6(46.9-96.2)	50±17.4(9-78.8)	**<0.001**
Time-in-range (daytime)(%)	76±11.5(48.8-97.4)	50.1±17.4(9.6-79.9)	**<0.001**
Time-in-range (night-time) (%)	75.9±16.7(38.9-93)	48.8 ± 20(6.5-77.7)	**<0.001**
Time in level 1 hypoglycemia(<3.9mmol/L) overall (%) (median)	2(0.2-28.6)	5.2(0.1-19.5)	0.056
Time in level 1 hypoglycemia(<3.9mmol/L) day-time (%) (median)	1.7(0-44)	4.4(0.15-18.5)	**0.039**
Time in level 1 hypoglycemia(<3.9mmol/L) night-time (%) (median)	1.8 (0-44)	4.1(0-31)	0.185
Time in level 1 hyperglycemia(>10mmol/L) overall (%) (median)	19.8(0-50)	37.1(4-73)	**<0.001**
Time in level 1 hyperglycemia(>10mmol/L) day-time (%) (median)	22.4 (0-49)	38.6 (4.2-71.6)	**<0.001**
Time in level 1 hyperglycemia(>10mmol/L) night-time (%) (median)	13.3 (0-60)	36.7 (1.6-77.4)	**0.001**

*CV* Coefficient of variation*, SD* Standard Deviation

However, the median time spent in level 1 hypoglycemia (< 3.9 mmol/L) overall and night-time were similar in the two groups.

**Fig. 2:**
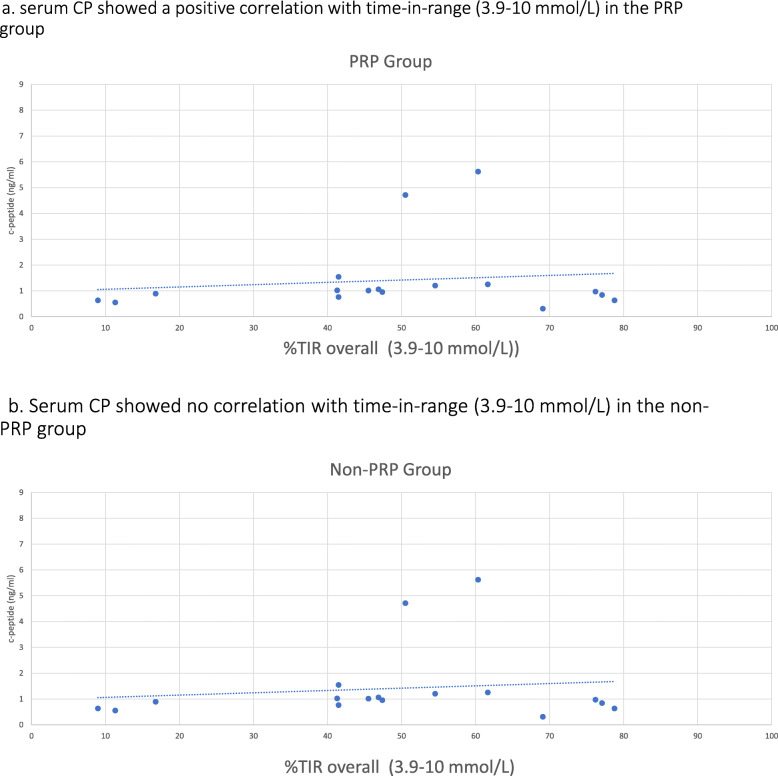
Correlation between C-peptide and TIR in PRP and non-PRP groups. **a** Serum CP showed a positive correlation with time-in-range (3. 9-10 mmol/L) in the PRP group. **b** Serum CP showed no correlation with time-in-range (3.9–10 mmol/L) in the non-PRP group

**Fig. 3:**
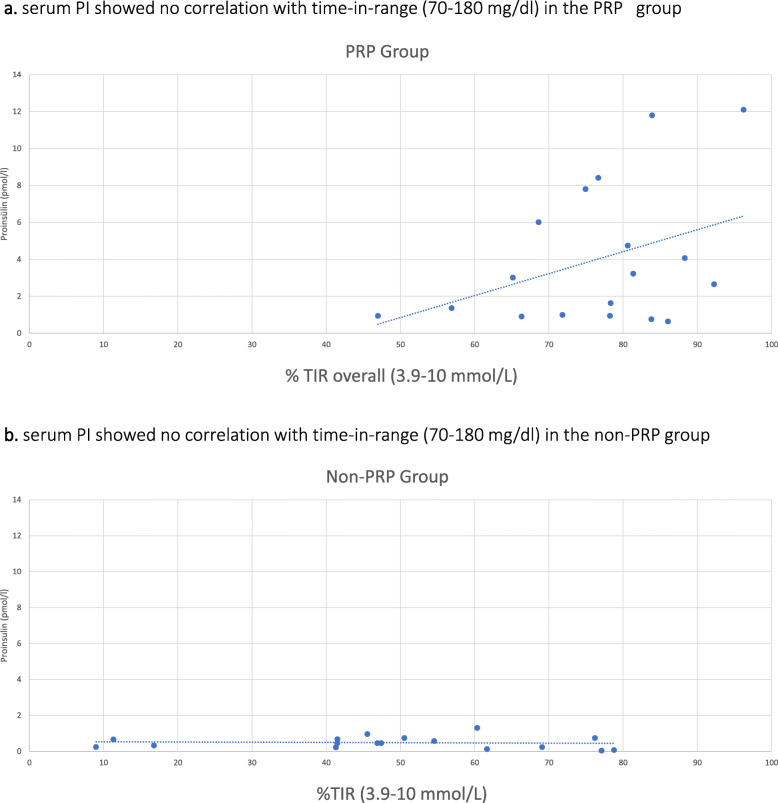
Correlation between Proinsulin and TIR in PRP and non-PRP groups. **a** Serum PI showed no correlation with time-in-range (3.9–10 mmol/L) in the PRP group. **b** Serum PI showed no correlation with time-in-range (3.9–10 mmol/L) in the non-PRP group

In correlation analyses (Table 3), the serum CP showed a positive correlation with time-in-range (3. 9-10 mmol/L) in the PRP group (Fig. 2), along with inverse correlations with the mean sensor glucose, mean sensor glucose SD and the median time spent in level 1 hyperglycemia. In the non-PRP group, serum CP was positively correlated with the time spent in level 1 hypoglycemia and level 1 hyperglycemia. However, serum PI showed no correlations with glycemic parameters in both groups (Fig. 3).

**Table 3:** Correlations between PI, CP, PI:CP ratio and glycemic parameters

		Time-in-range Overall	Time in Level 1 Hypoglycemia Overall	Time in Level 1 Hyperglycemia Overall	Mean HbA1C	Mean Sensor Glucose Overall	Mean SD Overall	Mean CV Overall
C- peptide	PRP	**r**_**1**_**:0.65 P**_**1**_**:0.03**	r_1_:-0.009 P_1_:0.97	**r**_**1**_**:-0.56 P**_**1**_**:0.015**	r_1_:0.27 P_1_:0.26	**r**_**1**_**:-0.47 P**_**1**_**:0.048**	**r**_**1**_**:-0.62 P**_**1**_**:0.006**	R_1_:-0.29 P_1_:0.23
	Non- PRP	r_2_: 0.09 P_2:_0.7	**r**_**2:**_**0.61 P**_**2:**_**0.009**	**r**_**2:**_**0.5** **P**_**2:**_**0.041**	r_2:-_0.06 P_2:_0.98	r_2:-_0.01 P_2:_0.95	r_2:_0.191 P_2:_0.462	R_2:_0.37 P_2:_0.14
Proinsulin	PRP	r_1_:0.23 P_1_:0.34	r_1_:-0.29 P_1_:0.24	r_1_:-0.16 P_1_:0.52	r_1_:0.16 P_1_:0.50	r_1_:-0.05 P_1_:0.84	r_1_:-0.24 P_1_:0.32	R_1_:-0.41 P_1_:0.08
	Non- PRP	r_2:_-0.94 P_2:_0.45	r_2:_0.07 P_2:_0.765	r_2:_0.24 P_2:_0.348	r_2:_0.16 P_2:_0.53	r_2:_0.16 P_2:_0.52	r_2:_0.22 P_2:_0.39	R_2:_0.02 P_2:_0.94
PI/CP ratio	PRP	r_1_:0.06 P_1_:0.78	r_1_:-0.38 P_1_:0.11	r_1_:0.01 P_1_:0.95	r_1_:0.15 P_1_:0.54	r_1_:0.09 P_1_:0.69	r_1_:-0.06 P_1_:0.79	R_1_:-0.42 P_1_:0.07
	Non-PRP	r_2:_-0.35 P_2:_0.62	**r**_**2:**_**-0.55 P**_**2:**_**0.02**	r_2:_0.09 P_2:_0.71	r_2:_0.28 P_2:_0.27	r_2:_0.37 P_2:_0.13	r_2:_0.18 P_2:_0.48	R_2:-_0.41 P_2:_0.09

*CV* Coefficient of variation*, SD* Standard Deviation*, PI:CP* Proinsulin to C-peptide ratio

## Discussion

Recent developments in continuous and intermittently scanned continuous glucose monitoring systems provide not only more efficient regulation of type 1 diabetes treatment, but also new information on glycemic parameters from the time of diagnosis. In this study, by using intermittently scanned glucose monitoring systems, the glycemic parameters of children with type 1 diabetes who are in RP and those who are not in RP were compared. As expected, the glycemic parameters in the RP were found to be closer to the target levels but were far from those of non-diabetic subjects [10]. It was found that while CP levels did have an effect on glycemic parameters during the PRP, there was no correlation between PI levels and glycemic parameters. In terms of glycemic parameters during the PRP, the CP level had a significant correlation with the TIR value.

New parameters are needed to define the PRP or preserved beta-cell reserve period. Although several studies [11–13] focused on the PRP and investigated PRP-related factors, data related to the glycemic parameters during the PRP are limited. Meng et al. examined the relationship between blood glucose fluctuations during various phases of diabetes and oxidative stress and showed that the mean glucose, glucose SD, the mean amplitude of glycemic excursions (MAGE) and incremental area under the blood glucose curve (IAUC) levels ​​during the PRP were lower than those during the acute metabolic disturbance and long-standing phases [14]. Similarly, in our study, the mean sensor glucose level, SD value, CV value, time-in-range, time in hyperglycemia were found to be lower in the PRP group, suggesting a better metabolic control (Table 2). While the mean CV of 32.3% was lower than the target value of 36% in the CGMS consensus, [6] the mean SD value was higher than that found in a recent study analyzing the CGMS data of healthy children [9]. In addition, time spent in level 1 hypoglycemia was found to be similar in the PRP vs. non-PRP groups, indicating that glycemic control in PRP is restricted.

CP is a useful and widely used method of assessing pancreatic beta-cell function. The formula for IDAA1c was derived using a higher CP cut-off value of 300 pmol/L. Therefore, although it previously played a role in defining the PRP, [3], it is no longer used to define the PRP. Venous blood CP levels can be measured in the random, fasting, or stimulated state. Random samples are taken at any time during the day without consideration of recent food intake, whereas fasting samples are taken after an 8 to 10 h fast [8]. In the present study, serum CP levels were measured in the random state, and as expected the mean CP level was significantly higher in the PRP group than in the non-PRP group. Although CP is a widely accepted biochemical parameter for pancreatic beta-cell reserve, data on the association between CP levels and glycemic parameters are very limited. In the present study, the presence of a correlation between CP levels and glycemic parameters was evaluated. In the PRP group, the mean CP level was inversely correlated with the mean sensor glucose and SD glucose whereas it positively correlated with TIR. These findings support that CP might be a relevant biochemical parameter in the PRP. However, the presence of a positive correlation between CP and time in both level 1 hypoglycemia and level 1 hyperglycemia in the non-PRP group suggests that CP is not a reliable parameter when it is low. On the other hand, release of islet hormones is regulated not only by direct actions of glucose and other nutrients, but also indirectly and potently by paracrine factors secreted by adjacent islet cells. The current knowledge shows that islet cells modulate each other’s secretory functions by very complex paracrine and even autocrine pathways. Low beta cell reserve, low CP may be associated with increased hypoglycaemia, possibly via secondary effects on the beta-cell alpha-cell cross talk and lower glucagon secretion [15, 16].

Recent studies reported an increased risk of developing 5-year T1D in antibody-positive first-degree relatives having an increased PI / CP ratio [17]. Similarly, the PI/CP ratio was found to be higher at the time of diagnosis of diabetes compared with the control group [18]. Although PI is used as a beta-cell stress marker, two recent studies showed that PI secretion was maintained for a long time and that the level of CP was still detectable many years after diagnosis in individuals with T1D [19, 20]. In our study, both the PI level and PI / CP ratio were significantly higher in the PRP group. In the non-PRP group, however, two children who were aged 6.2 and 12-year-old and whose diabetes duration was 2.3 and 8.7 years had CP levels below the measurable limit despite detectable PI levels. Due to the small size of the patient groups, it is difficult to comment on the differences in the maintenance of CP and PI secretions. In this study, no significant correlation was detected between proinsulin levels and glycemic parameters in any group, suggesting that proinsulin is not as clinically reliable as CP in the management of diabetes. Although its level in the PRP is higher than the non-PRP group, proinsulin shows beta cell stress and rather provides information about the earlier phase of diabetes [4] The PRP is a relatively later period of the beta cell injury process. Therefore, there may not be a relationship between glycemic parameters and proinsulin levels in this phase.

One of the main limitations of our study was the random measurement of CP and PI levels. These measures may have been affected by the degree of fasting, which was not taken into account because we recruited patients during scheduled outpatient follow-up visits. Measurements of stimulated CP and PI secretions using tests such as a mixed-meal tolerance test may be beneficial, as well. However, this study design would require more inconvenience to the participants and their families. Moreover, Watkins et al. conducted a research by random sampling for the CP and PI measurement in order to evaluate the β-cell function in persons with T1D [5] and Leighton et al. in their review article had stated that venous blood CP levels can be measured in the random, fasting, or stimulated state [8]. Another limitation was the lack of simultaneous blood glucose measurements with the use of isCGMS which does not require calibration. The accuracy of isCGMS was reported to be lower during hypoglycemia than during euglycemia and hyperglycemia [21]. However, time spent in level 1 hypoglycemia was not high and similar in both groups. Despite these limitations, we feel that this pilot study is important as it is the first study to evaluate glycemic parameters in the PRP with the use of isCGMS data and to examine the relationship between these parameters and Beta-cell reserve markers CP and PI. There is no doubt that a future study with larger patient numbers would show more generalizable results.

## Conclusions

In conclusion, although the glycemic profile during the PRP was better than that in the non-PRP group, the glycemic variability during this period was not as low as expected. It is also important to continue efforts to improve and maintain metabolic control during the PRP. Subcutaneous insulin infusion or automated insulin delivery systems may help to achieve better glycemic control in the remission phase as well. While the CP levels showed an association with TIR during the PRP, there was no correlation between PI levels and glycemic parameters. Further studies are needed to determine if PI might prove to be a useful parameter in clinical follow-up.

## Data Availability

The datasets used and/or analysed during the current study are available from the corresponding author on reasonable request.

## Abbreviations

CP: C-peptide
PI: Proinsulin
T1D: Type 1 Diabetes
isCGMS: Intermittently scanned continuous glucose monitoring systems
RP: Remission Phase
PRP: Partial Remission Phase
HbA1c: Hemoglobin A1C
IDAA1c: Insulin dose-adjusted hemoglobin A1c
CGMS: Continuous glucose monitoring systems
SD: Standard Deviation
CV: Coefficient of Variation
MAGE: The Mean amplitude of glycemic excursions
IAUC: Incremental area under the blood glucose curve
